# Experimental data on the removal of phenol by electro-H_2_O_2_ in presence of UV with response surface methodology

**DOI:** 10.1016/j.mex.2019.05.004

**Published:** 2019-05-09

**Authors:** Mohammad Malakootian, Alireza Nasiri, Mehrdad Khatami, Hakimeh Mahdizadeh, Pouria Karimi, Mohammad Ahmadian, Nastaran Asadzadeh, Mohammad Reza Heidari

**Affiliations:** a Environmental Health Engineering Research Center, Kerman University of Medical Sciences, Kerman, Iran; b Department of Environmental Health, School of Public Health, Kerman University of Medical Sciences, Kerman, Iran; c Department of Environmental Health Engineering, School of Public Health, Bam University of Medical Sciences, Bam, Iran; d NanoBioElectrochemistry Research Center, Bam University of Medical Sciences, Bam, Iran; e Students Research Committee, Kerman University of Medical Sciences, Kerman, Iran

**Keywords:** Electrode, Phenol, UV, Peroxide hydrogen

## Abstract

Phenol is classified as priority pollutant. Phenol and its derivatives are stable in water, environmental contamination, and health concerns that are used as raw material in many chemical industries. This study investigated the removal of phenol by electro-H_2_O_2_/UV system.

The response surface methodology (RSM) using central composite design (CCD) was used to modeling and optimization of experimental parameters such as pH, contact time, initial concentration of phenol, concentration of hydrogen peroxide, and current density.

The obtained results demonstrated that the efficiency of the electro-H_2_O_2_/UV system was maximum (>99%) under the optimal conditions for the phenol removal from aqueous solutions, 2 mM of hydrogen peroxide concentration, 50 mg/L of initial phenol concentration, pH of 5, 10 mA/cm^2^ of current density, reaction time of 25 min and 2.1 kW h/m^3^ of energy consumption. Therefore, the electro-H_2_O_2_/UV system is an efficient method for the removal of organic compounds from industrial wastewater.

## Graphical abstract

**Figure:**
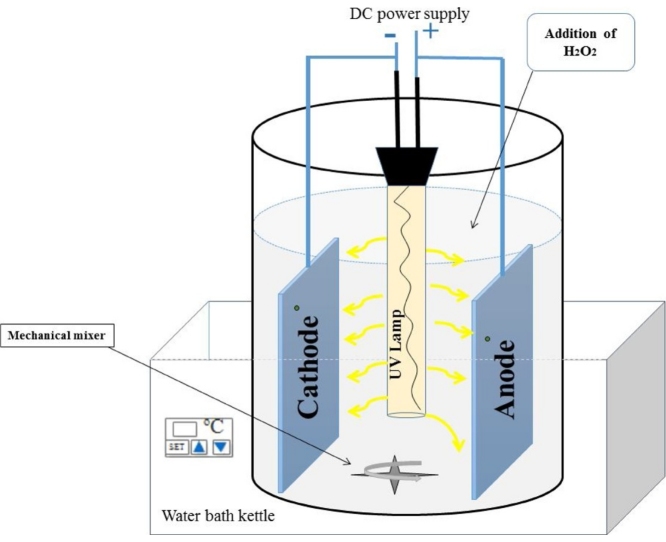

**Specifications Table**

**Table:** 

Subject Area:	*Environmental Science*
More specific subject area:	*Electrochemical, Advance Oxidation*
Protocol name:	*A combined system from Electrochemical and Advance Oxidation Process*
Reagents/tools:	H_2_O_2_, FeCl_3_.6H_2_O, CoCl_2_.6H_2_O, HNO_3_, NaOH, Tert-Butyl Alcohol, and chloroform (CHCl_3_) were purchased from Merck. Co, Germany. *UV– vis spectrophotometer (Shimadzu, Japan).*
*Experimental design:	*All removal experiments were bench scale that was done in a reactor 1 L, equipped with two electrodes aluminum and three UV lamps (6 W, Philips). Influences of pH (3-11), contact time(0-40 min), initial concentration of phenol (10-100 mg/L), concentration of hydrogen peroxide (0-4 mM), and current density (0-30 mA/cm2) in the Electro-H2O2/UV process on removal efficiency of phenol and COD were evaluated using central composite design (CCD). The concentration of phenol was determined by a UV– vis spectrophotometer (Shimadzu, Japan).*
Trial registration:	- Not applicable
Ethics:	Not applicable
*Value of the Protocol:	• *The electro-H_2_O_2_/UV system is based on the formation of free radical (OH•) that intensifies in the presence of radiation UV*. • *These results showed that the following mechanisms occur in this system: electrophoresis and aggregation, formation of a precipitate of pollutant, formation of a hydroxide for bonding to the pollutant, sweep coagulation in solution, oxidation to less toxicity, and removal of pollutant through its adhesion to bubbles*. • *By using a practical system of electro-H_2_O_2_/UV, > 98% of phenol and COD were removed from the aqueous solution*. • *The obtained data shows electro-H_2_O_2_/UV system is appropriate system for organic contaminate removal from industrial wastewater*.

## Description of protocol

### Data

This brief data set described the effectiveness of electro-H_2_O_2_/UV system in phenol removal from the aqueous solution. Table1 shows that levels of independent variables and experimental range in central composite design (CCD) were used as a response surface method for the optimization of electro-H_2_O_2_/UV system. The ANOVA test used for the quadratic modeling of phenol removal is presented in Table 1.

**Table 1:** ANOVA test for quadratic model.

Source	Sum of squares	Degree of freedom	Mean square	F value	P-value Prob > F	
Model	610.60	17	35.92	116.10	< 0.0001	significant
A-pH	310.23	1	310.23	1002.82	< 0.0001	significant
B-Time	34.25	1	34.25	110.70	< 0.0001	significant
C-Current density	64.59	1	64.59	208.78	< 0.0001	significant
D-Initial phenol	24.41	1	24.41	78.89	< 0.0001	significant
E-H_2_O_2_	149.26	1	149.26	482.49	< 0.0001	significant
AB	2.53	1	2.53	8.18	0.0074	significant
AC	0.28	1	0.28	0.91	< 0.0001	significant
AD	0.28	1	0.28	0.91	< 0.0001	significant
AE	0.78	1	0.78	2.53	< 0.0001	significant
BC	0.78	1	0.78	2.53	< 0.0001	significant
BD	0.031	1	0.031	0.10	< 0.0001	significant
BE	5.28	1	5.28	17.07	0.0002	significant
CD	1.53	1	1.53	4.95	0.0333	significant
CE	3.78	1	3.78	12.22	0.0014	significant
DE	0.031	1	0.031	0.10	< 0.0001	significant
A^2^	3.52	1	3.52	11.49	0.0020	significant
B^2^	7.49	1	7.49	24.47	< 0.0001	significant
Residual	8.88	29	–	–	–	–
Lack of Fit	8.00	22	0.36	2.91	0.0757	not significant
Pure Error	0.88	7	0.13	–	–	–
Cor Total	620.50	49	–	–	–	–
R-Squared	0.9857	–	–	–	–	–
Adj R-Squared	0.9758					
Pred R-Squared	0.9523					
Adequate Precision	44.003					

**Fig. 1:**
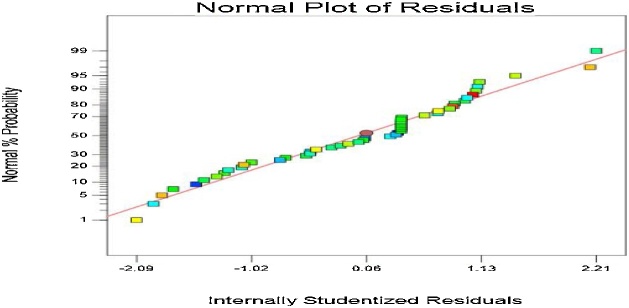
Normal probability plot of studentized residuals.

**Fig. 2:**
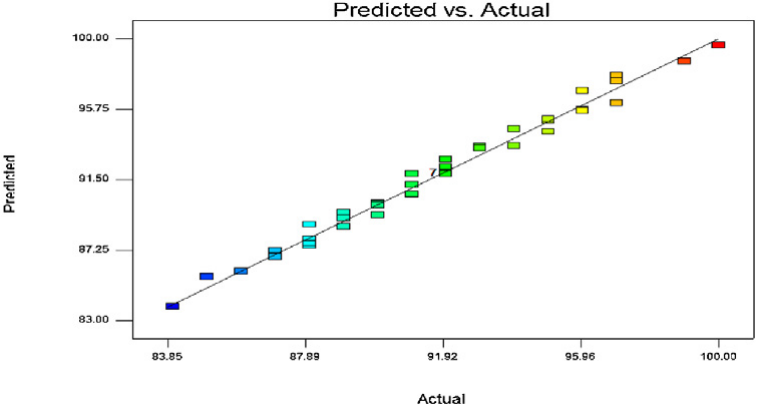
Predicted and actual data of phenol removal.

The normal probability plot of the studentized residuals and plot of the predicted versus actual on phenol removal efficiency are shown in Fig. 1, Fig. 2. A quadratic equation between dependent variable (phenol removal) and independent variables was obtained according to Eq. (1).

(1) Phenol Removal (%) = +91.79 - 2.68 A + 0.89 B + 1.22 C + 0.75 D + 1.86 E + 0.28 AB - 0.094 AC + 0.094 AD - 0.16 A E - 0.16 BC + 0.031 BD + 0.41 BE - 0.22 CD - 0.34 CE - 0.031DE + 0.26 A2 - 0.36 B2

**Fig. 3:**
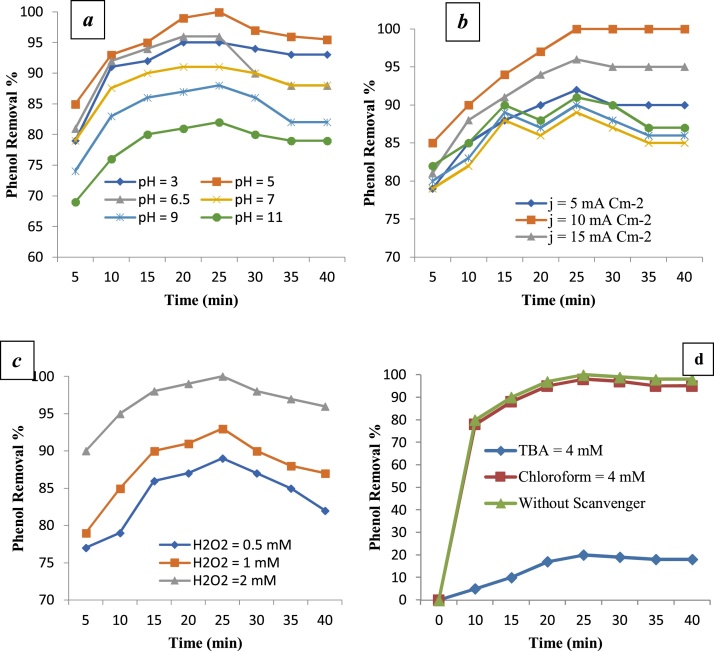
Effects of pH (a), current density (b), H_2_O_2_ concentration(c), and effects of radical scavengers (TBA and Chloroform) (d) on the removal efficiency of phenol.

**Fig. 4:**
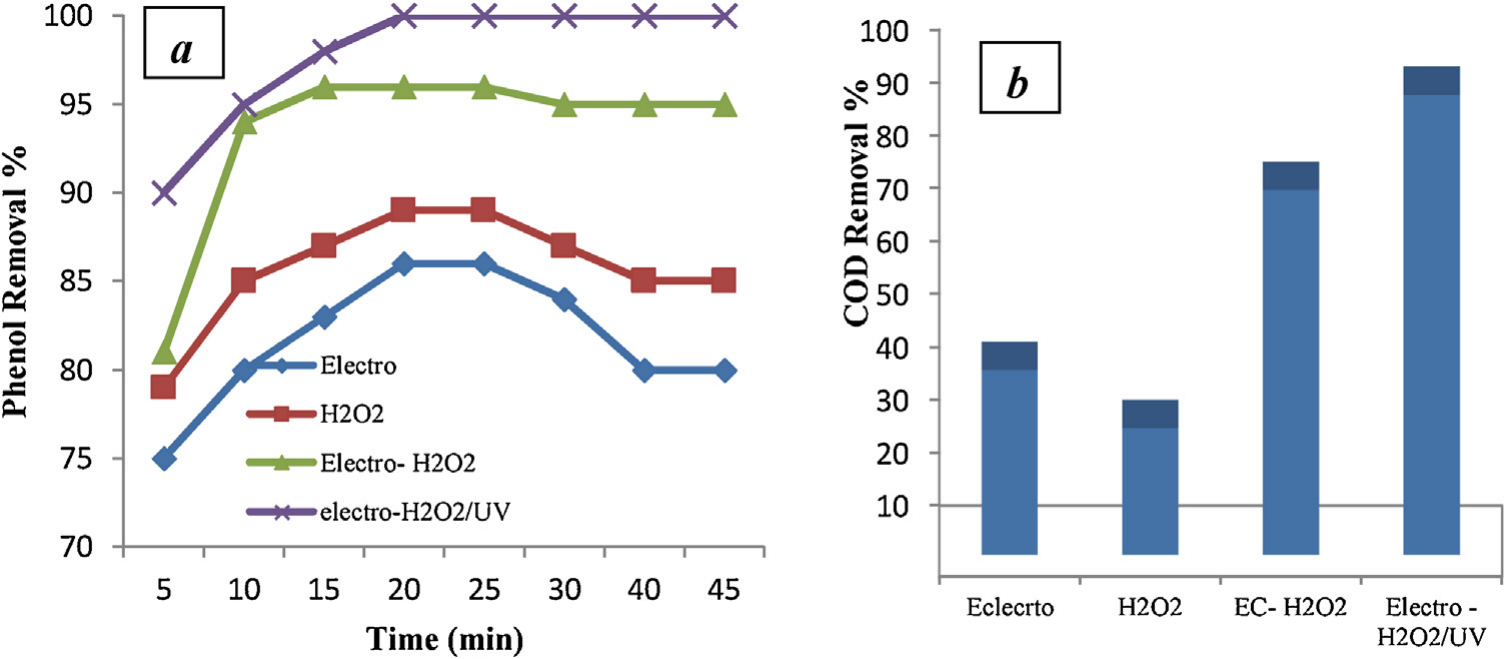
Phenol removal (a), COD removal (b) in different systems: H_2_O_2_ = 2.0 mM, j = 10 mAcm^−2^, and initial pH = 5.

Fig. 3 shows the effects of solution pH, H_2_O_2_ concentration, and radical scavengers (TBA and chloroform) on the removal efficiency of phenol, respectively. Fig. 4(a and b) demonstrates the removal efficiency of phenol and COD in different systems. In addiation, Table 2 shows the pseudo-first-order kinetic model for the removal efficiency of phenol by different systems.

**Table 2:** Phenol removal kinetic and pseudo - first-order rate constants values in different systems.

Removal process	K (min-1)	Linear coefficient (R^2^)
**Electrochemical**	0.0003	0.88
**H_2_O_2_**	0.004	0.85
**Electro -H_2_O_2_/UV**	0.0073	0.93

## Experimental design, materials, and methods

The electro-H_2_O_2_ reactor consisted of a 1.0 – L plexiglas vessel with two aluminum plate electrodes (1 mm thickness), in which the distance between the anode and cathode was 5 cm and the mode of electrode connection was bipolar to the DC power supply (current densities of 1–30 mA/cm^2^). One 30-W (UV-C) Mercury Lamp (Philips) in a quartz sheath at the reactor center that was fitted with an aluminum cover in a batch reactor was employed [1]. Specific amounts of Na_2_SO_4_ 0.1 M were added as the only supporting electrolyte [2]. Finally, hydrogen peroxide (0.5–4 mM) was added to the reactor. Then, the certain amount of hydrogen peroxide (0.5–4 mM) was added to the reactor, and a magnetic stirrer (400 rpm) was used in the reactor to maintain monotonous concentration at room temperature. pH meter and water bath temperature control system were used to maintain the reaction solution at the stable pH and temperature. The effect of pH (pH = 3–11) with 0.1 M HNO_3_ solution and 0.1 M NaOH solution was evaluated. All the experiments were 50 runs, the experiments designed by Design – Expert software (version7), based on central composite design (CCD), which was used to analyze three parameters such as pH (3–11), H_2_O_2_ dose (0.5–4 mM) and current density (1–30 mA/cm^2^) in phenol removal efficiency and removal optimum conditions [3]. The phenol and COD concentrations were determined using the 4-aminoantipyrine method and the dichromatic closed reflux method, respectively and according to the standard methods. H_2_O_2_, FeCl_3_.6H_2_O, CoCl_2_.6H_2_O, HNO_3_, NaOH, tert alcohol, and chloroform (CHCl_3_) were purchased from Merck, Germany. All the analyses were replicated at least 3 times, and the graphs and the respective error bars were plotted [4]. The percentage of COD and phenol removed was calculated as follows (Eq. (2)):

(2) $$\text{R}\;=\frac{\left[\text{i}\text{n}\text{p}\text{u}\text{t}\right]-[\text{o}\text{u}\text{t}\text{p}\text{u}\text{t}]}{\left[\text{i}\text{n}\text{p}\text{u}\text{t}\right]}\times \;100$$

The model equation in E shows k (min^−1^) and q_e_ and q_t_ (mgg^−1^) are a constant rate, the adsorption capacity at time t, and the equilibrium of pseudo- first order kinetics. The fit of experimental data to the kinetic model was assessed by the correlation coefficient (R_2_) and the residual root mean square error (RMSE). The value of R_2_, which might vary between 0 and 1, indicates the degree of fit of experimental data to the model [1]. The R_2_ expression is given by Eq. (3):

(3) $$\text{R}2\;=\frac{\sum_{\text{i}=1}^{\text{N}}{\left({\text{q}}_{\text{e}}-\;{\text{q}}_{\text{e},\text{e}\text{x}\text{p}}\right)}^{2}}{\sum_{\text{i}=1}^{\text{N}}{\left({\text{q}}_{\text{e}}-{\text{q}}_{\text{e},\text{e}\text{x}\text{p}}\right)}^{2}+\;{\left({\text{q}}_{\text{e}}-\;{\text{q}}_{\text{e},\text{e}\text{x}\text{p}}\right)}^{2}}$$

RMSE represents the match between the experimental data and the calculated data used for plotting the kinetic model, where n is the number of data points. It is defined as (Eq. (4)):

(4) $$\text{R}\text{S}\text{M}\text{E}\;=\sqrt{\frac{1}{\text{n}-2}\;\sum_{\text{i}=1}^{\text{N}}{\left({\text{q}}_{\text{e}}-\;{\text{q}}_{\text{e},\text{e}\text{x}\text{p}}\right)}^{2}}$$

Therefore, electric energy consumption is calculated as (Eq. (5)):

(5) $$\text{E}\;=\frac{\text{U}\text{I}{\text{t}}_{\text{E}\text{C}}}{\text{V}}$$

where E is the electrical energy [5], U is the cell voltage (V), I is the current density (A) and t EC is the time of the electro-H_2_O_2_/UV system per hour [6]. According to the results the minimum energy consumption was 2.15 kW h/kg.

As shown in Fig. 3, Fig. 4, the maximum efficiency of removal phenol and COD under optimum condition (2 mM of H_2_O_2_ concentration, 50 mg/L of initial phenol concentration, pH = 5, j = 10 mA/cm2, t =25 min, and 2.1 kW h/m3 of energy consumption) was 99% for phenol and 97% for COD.

Similar results in other research have been reported metronidazole removal by the combined system coupling an electro-Fenton process and conventional biological treatment [7,8], treatment of retting flax wastewater by Fenton oxidation and granular activated carbon [8], treatment of distillery industrial effluent by combining electrocoagulation with advanced oxidation processes [9]. This trend suggests that the presence of UV radiation has had a positive effect on the phenol removal efficiency [10]. The results in Table 1 indicate that the removal behavior of the contaminant over time follows pseudo-second-order models, in accordance with the results obtained by Seid Mohammadi [11].

## Funding sources

This paper is the result of the approved project at Kerman University of Medical Sciences.
